# Computational modelling of cancerous mutations in the EGFR/ERK signalling pathway

**DOI:** 10.1186/1752-0509-3-100

**Published:** 2009-10-05

**Authors:** Richard J Orton, Michiel E Adriaens, Amelie Gormand, Oliver E Sturm, Walter Kolch, David R Gilbert

**Affiliations:** 1 Institute of Comparative Medicine, Faculty of Veterinary Medicine, University of Glasgow, Glasgow, G61 1QH, UK; 2 Bioinformatics Research Centre, Department of Computing Science, University of Glasgow, Glasgow, G12 8QQ, UK; 3 Department of Bioinformatics - BiGCaT, Maastricht University, Maastricht, The Netherlands; 4 Department of Experimental Medical Science, Faculty of Medicine, Lund University, Lund, Sweden; 5 St Jude Children's Research Hospital, Memphis, Tennessee, TN 38105, USA; 6 Beatson Institute for Cancer Research, Garscube Estate, Glasgow, G61 IBD, UK; 7 School of Information Systems, Computing and Mathematics, Brunel University, Uxbridge, Middlesex, UB8 3PH, UK

## Abstract

**Background:**

The Epidermal Growth Factor Receptor (EGFR) activated Extracellular-signal Regulated Kinase (ERK) pathway is a critical cell signalling pathway that relays the signal for a cell to proliferate from the plasma membrane to the nucleus. Deregulation of the EGFR/ERK pathway due to alterations affecting the expression or function of a number of pathway components has long been associated with numerous forms of cancer. Under normal conditions, Epidermal Growth Factor (EGF) stimulates a rapid but transient activation of ERK as the signal is rapidly shutdown. Whereas, under cancerous mutation conditions the ERK signal cannot be shutdown and is sustained resulting in the constitutive activation of ERK and continual cell proliferation. In this study, we have used computational modelling techniques to investigate what effects various cancerous alterations have on the signalling flow through the ERK pathway.

**Results:**

We have generated a new model of the EGFR activated ERK pathway, which was verified by our own experimental data. We then altered our model to represent various cancerous situations such as Ras, B-Raf and EGFR mutations, as well as EGFR overexpression. Analysis of the models showed that different cancerous situations resulted in different signalling patterns through the ERK pathway, especially when compared to the normal EGF signal pattern. Our model predicts that cancerous EGFR mutation and overexpression signals almost exclusively via the Rap1 pathway, predicting that this pathway is the best target for drugs. Furthermore, our model also highlights the importance of receptor degradation in normal and cancerous EGFR signalling, and suggests that receptor degradation is a key difference between the signalling from the EGF and Nerve Growth Factor (NGF) receptors.

**Conclusion:**

Our results suggest that different routes to ERK activation are being utilised in different cancerous situations which therefore has interesting implications for drug selection strategies. We also conducted a comparison of the critical differences between signalling from different growth factor receptors (namely EGFR, mutated EGFR, NGF, and Insulin) with our results suggesting the difference between the systems are large scale and can be attributed to the presence/absence of entire pathways rather than subtle difference in individual rate constants between the systems.

## Background

**Figure 1:**
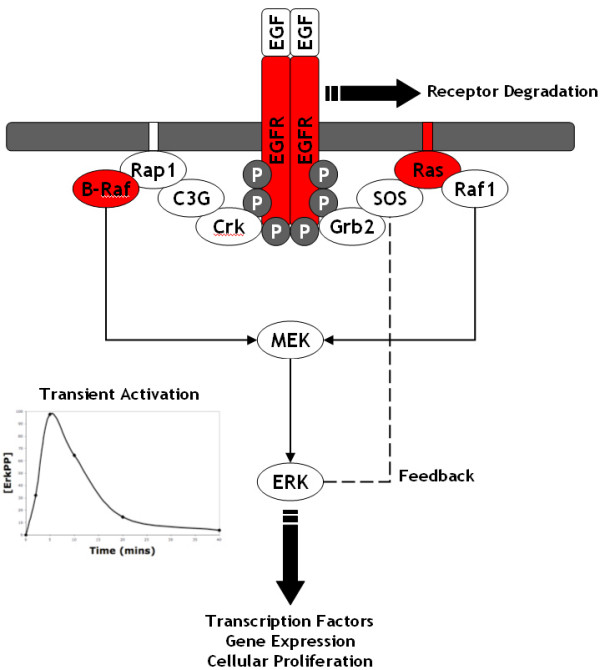
**Schematic of the EGF activated ERK pathway**: This is a schematic of the EGF activated ERK pathway beginning at the level of EGF binding to EGFR and finishing at the level of ERK; see the text for more details on the features and functions of the pathway. Mutations to the proteins highlighted in red are well known to result in the constitutive activation of ERK, leading to the subsequent development cancer.

Mitogen Activated Protein Kinase (MAPK) pathways are at the heart of molecular signalling networks that govern the growth, proliferation, differentiation and survival of many, if not all, cell types [1]. MAPK pathways are deregulated in various diseases ranging from cancer to immunological, inflammatory and degenerative syndromes, and thus represent increasingly important drug targets. Perhaps the most important and intensively studied MAPK pathway is the Extracellular-signal Regulated Kinase (ERK) pathway, which is typically initiated by the activation of cell surface receptors, such as the Epidermal Growth Factor Receptor (EGFR; Figure 1). The EGFR is a growth factor receptor that induces cell proliferation through the binding of its ligand, Epidermal Growth Factor (EGF). The binding of EGF to the EGFR induces conformational changes within the receptor that increases the catalytic activity of its intrinsic tyrosine kinase and also promotes the dimerisation of receptors. This dimerisation results in the auto-phosphorylation of numerous tyrosine residues in the receptor, which act as docking sites for a plethora of cytoplasmic adaptor proteins, typically containing SH2 or PTB domains. Among these adaptor proteins are Grb2 and Crk which are able to recruit the guanine nucleotide exchange factors SOS and C3G, respectively, to the receptor complex. This recruitment brings SOS and C3G into close proximity to their membrane bound targets, the small G-proteins Ras and Rap1, respectively, where they can load them with GTP. Ras-GTP and Rap1-GTP bind Raf kinases with high affinity translocating them from the cytoplasm to the cell membrane where they are activated. Active Raf proteins dual phosphorylate and activate MAPK/ERK kinase (MEK) which in turn dual phosphorylates and activates ERK. Activated ERK has over 100 targets in both the cytoplasm and nucleus [2], including numerous transcription factors, and can therefore directly effect gene expression and influence cellular outcome. In addition, ERK is able to phosphorylate SOS (via p90^Rsk^) which results in its dissociation from Grb2, thus forming a negative feedback loop within the pathway [3-7].

A common cell line used to investigate ERK signalling from growth factor receptors is the PC12 (rat pheochromocytoma) cell line. In PC12 cells, EGF stimulates a strong but transient activation of ERK, peaking at ~5 mins and returning to basal levels at ~30 mins, which leads to cellular proliferation [8,9]. In contrast, Nerve Growth Factor (NGF) stimulates a sustained activation of ERK leading to the neuronal differentiation of PC12 cells [8,9]. There is now compelling evidence that the duration of the ERK signal governs whether PC12 cells proliferate or withdraw from the cell cycle and differentiate into a neuronal phenotype [9,10]. Although the PC12 system has been well studied, it is still unclear how different ERK signal dynamics can be robustly controlled by different upstream receptors utilising the same pathway. However, there are currently a number of theories such as differences in the strength of feedback loops between receptor systems [11,12] and differences in the adaptor proteins that can bind to the receptor to initiate the ERK pathway [9]. Numerous other growth factor receptors (such as the Insulin, Fibroblast, and Platelet-Derived receptors) also use this same ERK pathway to generate different signals and different cellular responses. Therefore, an understanding of the critical differences between the receptor systems and how they utilise the same pathway to generate different responses would be a major advance.

Alterations in the cellular genome affecting the expression or function of genes controlling cell growth are considered to be the main cause of cancer [13]. Common alterations include mutations to the Ras and B-Raf proteins as well as mutation or overexpression of the EGFR, which all lead to the constitutive activation of ERK. Approximately 30 % of all human cancers contain a mutation to one of the *ras *oncogenes (Ki-*ras*, Ha-*ras*, N-*ras*) that causes the resulting protein to be constitutively active [13]. A constitutively active Ras is able to continually activate Raf kinases and therefore MEK which subsequently results in the constitutive activation of ERK and uncontrolled cellular proliferation. Constitutively active Ras is typically caused by mutations that prevent GTP hydrolysis, thus locking Ras in a permanent 'on' state. One of the most common Ras mutations is a glycine to valine mutation at residue 12 (Ras^V12^) which renders Ras insensitive to inactivation by Ras-GAP and thus locked in the 'on' state. Somatic missense mutations of B-Raf have been reported in 66 % of malignant melanomas and at lower frequencies in a wide range of other human cancers [14]. By far the most common mutation is a single substitution of glutamic acid to valine at residue 600 (B-Raf^V600E^) which greatly elevates the kinase activity of B-Raf and results in the constitutive activation of ERK *in vivo*, independent of Ras [14].

Mutations, deletions and overexpression resulting in constitutive activation of EGFR have long been associated with various types of cancer [15]. The most common mutation of the EGFR found in human cancer is EGFRvIII which has been found in more than 50 % of high and low grade gliomas [16] and in 21 of 27 breast carcinomas [17,18], amongst others. EGFRvIII can be caused by intragene rearrangements or alternative splicing resulting in the loss of domains I and II from the extracellular domain. EGFRvIII has a constitutively activated tyrosine kinase which results in the phosphorylation of receptor tyrosine residues and the continual recruitment of adaptor proteins, and subsequently the constitutive activation of ERK and uncontrolled cellular proliferation, independent of ligand interaction. It has also been shown that EGFRvIII is not internalised [19], thus avoiding the receptor degradation pathway, and is often amplified resulting in overexpression [17,20]. Another EGFR deletion is EGFRvI which is a total deletion of the extracellular domain resulting in a constitutively active EGFR which resembles the *v-erb*-B oncoprotein [20,21]. Gene amplification of the EGFR gene has also been observed in a number of different tumours and found to be present in ~40 % glioblastoma multiforme [22]. Overexpression of EGFR was also frequently observed in breast, bladder, cervix, kidney, and ovarian tumours [23] as well as in lung cancer and various squamous carcinomas [15]. Overexpression results in a greatly increased number of receptors on the cell membrane. This means that receptors randomly bump into each other with high frequency enabling them to dimerise and auto-phosphorylate in the absence of ligand and thus leads to the constitutive activation of the ERK pathway; although these receptors are degraded along the normal degradation pathway [24], they would be quickly replenished due to the overexpression.

Over recent years the computational modelling of biological systems has become increasingly valuable and there are now a wide variety of models of the ERK pathway available which have led to some novel insights and interesting predictions as to how this system functions [1]. Early models of the ERK pathway were focussed on investigating the properties and behaviour of the core cascade itself. For example, [25] showed that the ERK cascade exhibited ultrasensitivity whilst [26,27] showed that the activating dual phosphorylation of ERK was accomplished via a two-collision distributive mechanism. Nowadays, models routinely incorporate receptors and the plethora of adaptor proteins which can bind to them and activate the core ERK cascade. These models have been used to investigate various aspects of the biological behaviour of this system such as the role of negative feedback [11] and receptor internalisation [28] as well as the temporal dynamics of activation by different receptors [29,30].

In this study, we have used computational modelling techniques to investigate what effects various cancerous alterations have on signalling through the ERK pathway. We have generated a new model of the EGF activated ERK pathway which was based on a previously published model by [29] and has been verified by our own experimental data. We then altered our model to represent various cancerous situations such as Ras, B-Raf and EGFR mutations causing constitutive activation, as well as EGFR overexpression. Analysis of the models showed that different cancerous situations resulted in different signalling patterns through the ERK pathway, especially when compared to the normal EGF signal pattern. Our results suggest that different routes to ERK activation are being utilised in different cancerous situations, which therefore has interesting implications for drug selection strategies. Furthermore, our model also highlights the importance of receptor degradation in normal EGF signalling, and suggests that receptor degradation is a key difference between the signalling from different growth factor receptors - specifically the EGF and NGF receptors. Detailed information on the model as well as our analysis results is presented below.

## Methods

**Figure 2:**
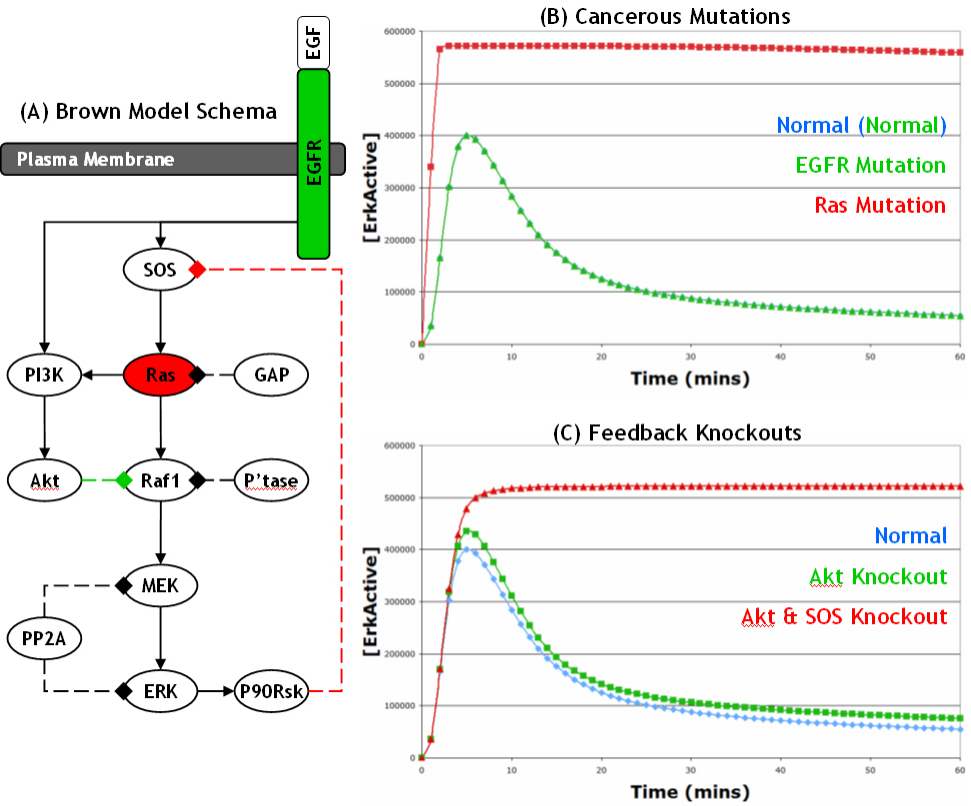
**Schematic and simulations of original Brown et al. (2004) EGF model**: (A) This is a basic schematic of the original model of the EGF activated ERK pathway developed by [29]; activating reactions are shown with solid lines ending in arrows whereas deactivating reactions are shown with dashed lines ending in diamonds. We used this model to investigate the effects of a Ras mutation (highlighted in red) and an EGFR mutation (green), as well as the roles of the SOS (highlighted with red line) and Raf-1 (green) negative feedback loops. All the lines in graph (B) and (C) represent simulated active ERK levels over 60 minutes. (B) The blue line represents active ERK levels from the normal EGF model, whereas the green and red lines represent active ERK levels from the Ras and EGFR mutation models, respectively; the EGFR mutation (green line) is practically indistinguishable from the normal simulation (blue line) and hence the blue line is obscured from view. (C) The blue line is the same as in (B), whereas the green line represents active ERK levels after the Raf-1 feedback loop has been knocked out, and the red line represents active ERK levels after the Akt feed-forward and SOS feedback loops have been knocked out.

In 2004, Brown *et al*. [29] developed computational models of the EGF and NGF activated ERK pathway in PC12 cells. [29] initially constructed the topological structure of the model and then used a novel ensemble method to automatically assign values to model parameters based on available experimental time course data. Using this approach, [29] generated models of the EGF and NGF activated ERK pathway and used them to make a number of interesting predictions; for example, that knocking out Akt would have little effect on ERK activation. Overall, the Brown EGF model consists of 13 different protein species involved in 16 biochemical reactions, which primarily utilise Michaelis-Menten kinetics, and considers the SOS-Ras-Raf-1 pathway leading to ERK activation as well as the SOS negative feedback and Akt negative feed-forward loops (Figure 2a).

In this study, we were interested in investigating what effects various cancerous alterations had on signalling through the EGF activated ERK pathway. Initially, we took the original Brown EGF model (downloaded from BioModels [31]) and investigated what effects introducing a Ras or an EGFR mutation had on ERK signalling (Figure 2a); to accomplish this, the software tool COPASI [32] was used for the construction, simulation and analysis of models. Under normal conditions, the original Brown model correctly predicts that EGF stimulates the transient activation of ERK (Figures 2b). Furthermore, when Ras is mutated causing it to be constitutively active, the model correctly predicts that ERK is also constitutively activated (Figure 2b). However, when EGFR is mutated causing it to be constitutively active, the Brown model incorrectly predicts that ERK is only transiently activated (Figure 2b). This is certainly incorrect because, as described above, mutations in EGFR that cause it to be constitutively active are well known to lead to the constitutive activation of ERK and to the subsequent development of cancer. After brief investigations, we found that the reason for this incorrect prediction was that the negative feedback loop from active ERK to SOS (via p90Rsk) is very strong and rapidly shuts down the Ras to ERK signalling pathway, resulting in only a transient activation of ERK despite the constitutive activation of EGFR. Deleting the SOS feedback loop from the model results in a sustained activation of ERK after normal EGF stimulation which suggests that it is the key process involved in terminating the signal from the EGF receptor in the Brown model (Figure 2c); in contrast, deleting the Akt to Raf-1 feed-forward loop has little effect on the ERK signal (Figure 2c). The ERK to SOS negative feedback loop has been well characterised [3-7,33] and there is therefore little doubt that it does exist. However, our results here strongly suggest that due to this feedback loop, an alternative to the SOS-Ras-Raf-1 pathway must exist in order for mutated EGFR to constitutively activate ERK. In addition, making EGFR constitutively active made little difference to the model behaviour because, in the Normal Brown EGF model, all of the receptors are very rapidly activated by EGF and they remain activated because the degradation of receptors is not taken into account. Thus, all normal EGFR are essentially constitutively active after stimulation with EGF and hence there is little difference between the normal and EGFR mutation model simulations. This highlights the potential importance of the process of receptor degradation, as without it normal EGFR receptors remain constitutively active.

**Figure 3:**
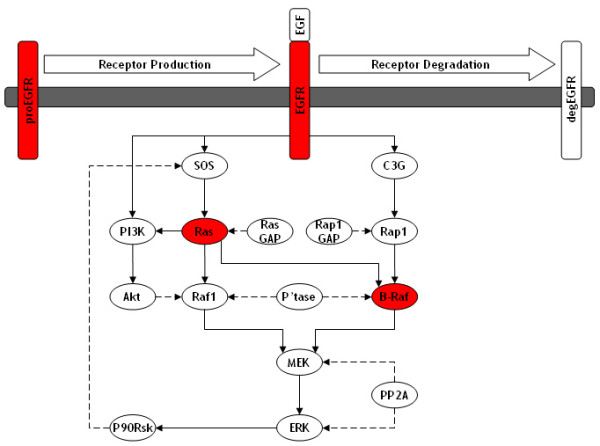
**Schematic of new model of EGF activated ERK pathway**: This is a schematic of our new model of the EGF activated ERK pathway which was developed from the original [29] EGF model. The new model considers receptor production and degradation as well as the Ras and Rap1 pathways leading to the activation of ERK. We used this model to investigate the effects of a number of cancerous alterations such as a Ras mutation, B-Raf mutation, EGFR mutation, and EGFR overexpression; all these cancerous alterations are highlighted in red.

As EGFR mutations are well known to constitutively activate ERK causing cancer, we decided to generate a new model of the EGF activated ERK pathway which would correctly predict the effects of various cancerous alterations and enable further investigations. To accomplish this we took the original Brown EGF model [29,33] and made a number of modifications and expansions. These modifications were informed by the scientific literature and in particular by the recent study by [9] which is one of the most comprehensive and up-to-date studies of the EGF and NGF signalling pathways in PC12 cells. In this study, [9] reported that EGF stimulation results in the activation of both Ras (via SOS recruitment) and Rap1 (via C3G recruitment); however, the original Brown EGF model did not include the C3G/Rap1 pathway. [9] also reported that activated Ras is able to activate both Raf-1 and B-Raf, adding additional complexity to the pathway, whilst activated Rap1 is only able to activate B-Raf. Furthermore, [9] reported that EGFR was rapidly ubiquitinated and subsequently degraded after EGF stimulation. Therefore, we developed a new model of the EGF activated ERK pathway by using the original Brown EGF model as a base to develop from. Our new model consists of 17 proteins involved in 31 reactions, which utilise primarily Michaelis-Menten but also mass action kinetics, and considers the production and degradation of EGFR as well as the Ras and Rap1 pathways leading to ERK activation (Figure 3); a Systems Biology Markup Language (SBML; [34]) version of our model is available with this publication (see Additional file 1) or at the following website: . We firstly validated the behaviour of our model against our own laboratory data of EGF stimulated ERK activation in PC12 cells and then used the model to investigate the effects of various cancerous alterations on the ERK signalling pathway; specifically, the effects of mutations in Ras, B-Raf and EGFR and also the effects of EGFR overexpression.

## Results and Discussion

### EGFR Signalling

**Figure 4:**
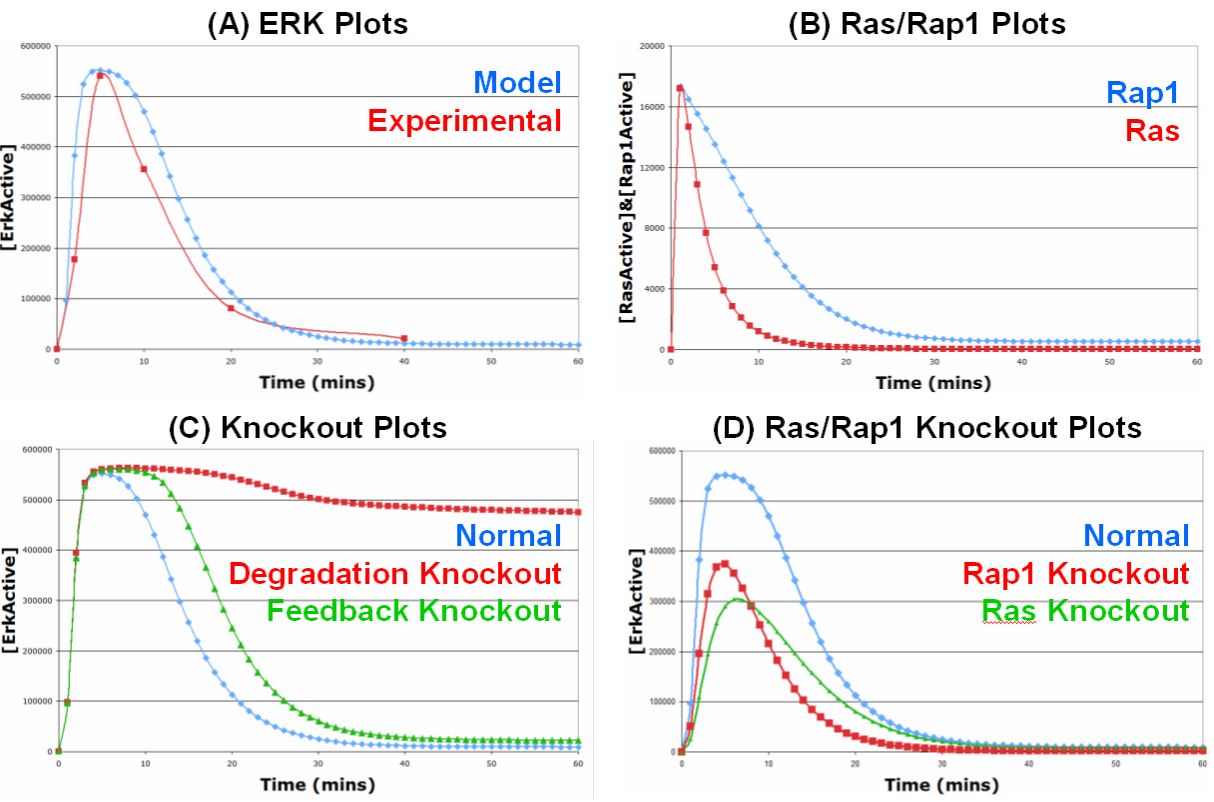
**Simulation results of our new EGF activated ERK pathway model**: (A) ERK Plots: The blue line represents the simulated levels of active ERK over 60 minutes from the model whereas the red line represents the experimentally measured levels of active ERK over 40 minutes from the laboratory; as the lab data is qualitative rather than quantitative, it has been rescaled for comparison to the simulation data. (B) Ras/Rap1 Plots: The red and blue lines represent the simulated levels of active Ras and active Rap1, respectively, over 60 minutes from the model. (C) Knockout Plots: All the lines on this graph represent simulated active ERK levels over 60 minutes. The blue line is the same as in (A). The green line represents active ERK levels when the SOS negative feedback loop is knocked out, whereas the red line represents active ERK levels when receptor degradation is knocked out. (D) Ras/Rap1 Knockout Plots: All the lines on this graph represent simulated active ERK levels over 60 minutes. The blue line is the same as in (A). The green line represents active ERK levels when Ras is knocked out, whereas the red line represents active ERK levels when Rap1 is knocked out.

Firstly, we simulated our new model under normal EGF stimulation conditions to verify that it still gave a strong transient activation of ERK. As can be seen in Figure 4a, ERK is rapidly activated reaching a maximum at ~5 mins and returning to basal levels at ~30 mins. We validated the behaviour of the model by comparing it to our own experimental data of EGF stimulated ERK activation in PC12 cells and there was a good fit between the model and experimental data (Figure 4a); please see the Additional file 2 for a description of laboratory experiment protocols. As can be seen, both the model and the experimental data have similar shapes, peak at the same time point, and return to basal levels at the same time. As there are two pathways leading from the receptor to ERK activation, specifically the Ras and Rap1 pathways, we investigated if the normal EGF system was predominantly using one pathway or using both equally. As can be seen in Figure 4b, the EGF system uses both the Ras and Rap1 pathways almost equally (which conforms well with the observations of [9]), but the Ras signal is terminated quicker due to the presence of the ERK to SOS (via P90Rsk) negative feedback loop. The relative contribution of the Ras and Rap1 pathways was investigated further through knockout experiments (Figure 4d). As can be seen, knocking out either Ras or Rap1 has similar effects on the activated ERK curve, with both knockouts resulting in a similar lowering of the peak ERK activation. However, knocking out Ras results in a slightly lower peak ERK signal when compared to the Rap1 knockout, this is probably to be expected given that Ras can activate both Raf-1 and B-Raf whilst Rap1 can only activate B-Raf. On the other hand, knocking out Rap1 results in a signal of shorter duration when compared to the Ras knockout, which is again to be expected given that Rap1 remains active for longer (Figure 4b) as there is no negative feedback loop within the Rap1 pathway. This suggests that the Ras pathway primarily contributes to the peak of the ERK signal, whilst the Rap1 pathway contributes to the peak (although less than Ras), as well as the duration of the ERK signal. What is important here is that the EGFR system does not appear to favour either or the two pathways and uses both relatively equally, this is important for comparison in the next section when cancerous mutations are introduced.

One interesting point is that the SOS negative feedback loop is no longer essential for efficient signal shutdown and the transient activation of ERK, as deleting it has only a slight effect on the ERK signal (Figure 4c). Instead, receptor degradation is now the key factor in signal termination as deleting the process of receptor degradation results in the sustained activation of ERK (Figure 4c). This is a decisive improvement over the original Brown EGF model, where the SOS negative feedback loop was found to be essential for signal termination and the transient response (Figure 2c), as receptor degradation was not considered. In our model, receptor degradation is now essential because there is no negative feedback loop present on the C3G/Rap1/B-Raf pathway so the signal has to be shutdown at the receptor level to achieve a transient response, which makes the process of receptor degradation essential. This has interesting and important implications for signalling from other growth factor receptors which are discussed further in the next section.

A sensitivity analysis of the EGFR model was also performed to identify the reactions that have the most influence on the ERK signal (see Additional file 2, Figure S6; and see Additional file 3 for model parameter values and sources). Overall, reactions contained within the Rap1 pathway were found to be more sensitive than the corresponding reactions in the Ras pathway. This is to be expected given that the Ras pathway is contained within a strong negative feedback loop, thus reducing the sensitivities of the reactions contained within the loop. However, although less sensitive, the Ras pathway is still a key feature of the EGFR system. This is illustrated in the knockout plots in Figure 4d, as knocking out Ras has a greater effect on the peak of the ERK signal than knocking out Rap1. This again highlights the fact that the normal EGFR system utilises both of the pathways to relay its signal. The sensitivity analysis also highlighted EGF receptor degradation as one of the most sensitive reactions in the model. This further highlights the importance of the process of receptor degradation in addition to the knockout experiments in Figure 4c.

### Cancerous Mutations

**Figure 5:**
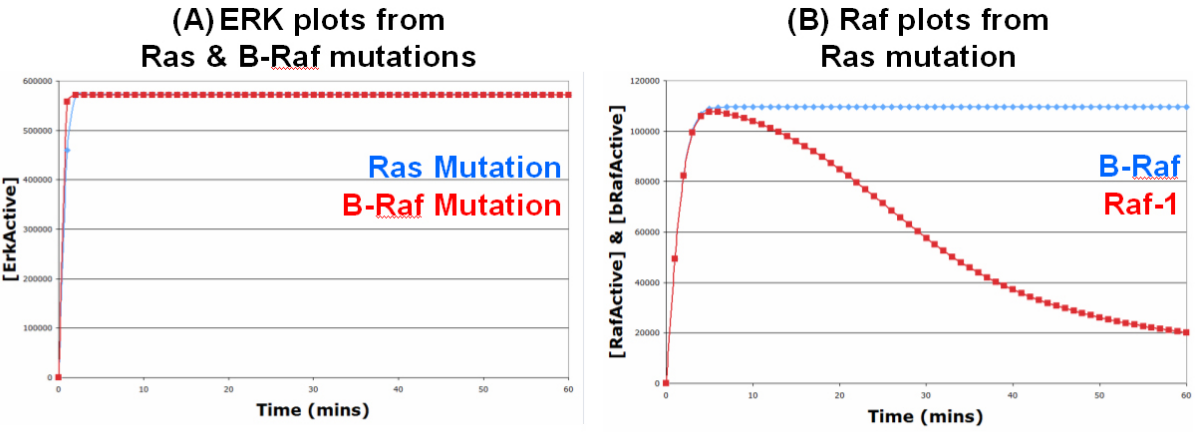
**Simulation results from Ras and B-Raf mutation models**: (A) Ras and B-Raf Mutations: The red and blue lines represent the simulated levels of active ERK over 60 minutes with a B-Raf and Ras mutation, respectively; as both lines are so similar, the red line obscures the blue line from view for the most part. As can be seen, both Ras and B-Raf mutations result in the constitutive activation of ERK to very high levels, as expected. (B) Raf Plots: Both the lines on this graph come from the Ras mutation model. The red and blue lines represent the simulated levels of active Raf-1 and B-Raf, respectively. As can be seen, the Ras mutation results in the sustained activation of B-Raf to very high levels, but the activation of Raf-1 is more transient and begins to decline due to the Ras/PI3K/Akt feed-forward loop.

Initially, we were interested in investigating what effects introducing various cancerous alterations had on signalling through the ERK pathway. Therefore, we introduced a mutated constitutively active Ras into the model and analysed its effects. In the absence of EGF, the mutated Ras resulted in the constitutive activation of ERK (Figure 5a), as expected. This is because a constitutively active Ras, which cannot be deactivated by Ras-GAP, is always able to activate Raf-1 and B-Raf which results in the constitutive activation of MEK and subsequently ERK to high levels. Due to the presence of the PI3K -> Akt -> Raf-1 feed-forward loop which deactivates Raf-1, the mutated Ras appears to signal predominately via B-Raf (Figure 5b). However, although a large proportion of the Raf-1 is deactivated via the PI3K/Akt pathway, active Raf-1 levels still remain high enough to fully activate ERK on its own if B-Raf were not present (see Additional file 2, Figure S3); this is because the Raf-1 feed-forward loop is relatively weak, especially when compared to the SOS feedback loop (Figure 2c). These results therefore suggest that drugs that target either B-Raf or Raf-1 individually will be ineffective in treating cancers caused by Ras mutation, whilst a drug (or combination of drugs) that can target both Raf isoforms will be much more effective. As expected, introducing a mutated constitutively active B-Raf into the model also resulted in the constitutive activation of ERK (Figure 5a). This is because a constitutively active B-Raf cannot be deactivated by its phosphatase and there is no negative feedback loop within the B-Raf/MEK/ERK module to terminate or hinder the signal. These results could not have been obtained from the original Brown EGF model as B-Raf and the link between Ras and B-Raf was not considered.

**Figure 6:**
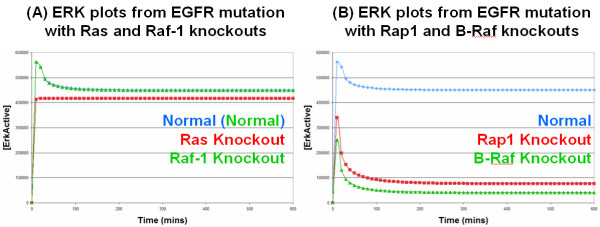
**Simulation results from EGFR mutation model**: All the lines in these graphs represent simulated active ERK levels over 600 minutes. (A) Ras and Raf-1 Knockouts: The blue line represents active ERK levels with an EGFR mutation, whereas, the red and green lines represent active ERK levels with an EGFR mutation but also with a Ras or Raf-1 knockout, respectively; the green line is practically identical to the blue line and hence obscures the blue line from view. (B) Rap1 and B-Raf Knockouts: The blue line is the same as in (A), whereas, the red and green lines represent active ERK levels with an EGFR mutation but also with a Rap1 or B-Raf knockout, respectively.

Our most interesting result came from introducing a mutated constitutively active EGFR into the model. Mutated, constitutively active, EGFR are able to activate both SOS and C3G in the absence of EGF. As the mutated EGFR does not bind EGF, it is not recognised as an active receptor by the cellular machinery and is therefore not degraded along the activated receptor degradation pathway [15,19,35]. However, there is natural degradation/turnover of unbound receptors (whether they are normal or mutated) which is represented in the model, but this is balanced out by the natural production of receptors, thus keeping a constant level of receptors on the membrane. Introducing the mutated EGFR into the model resulted in the constitutive activation of ERK, in the absence of EGF (Figure 6a), as expected; this is in direct contrast to the original Brown EGF model which incorrectly predicted that introducing a constitutively activated EGFR would have no effect on the transient ERK signal (Figure 2b). However, what is interesting is that mutated EGFR receptors appear to signal almost exclusively via the C3G/Rap1/B-Raf pathway. This can be effectively illustrated by knockout experiments, as knocking out Ras or Raf-1 has little effect on active ERK levels (Figure 6a) whereas knocking out Rap1 or B-Raf has a dramatic effect and reduces active ERK levels to almost basal levels (Figures 6b). This is in direct contrast to the pattern of signalling observed with normal activated EGFR receptors which signal equally through both the Ras and Rap1 pathways (Figure 4b). Further investigations showed that this was again due to the presence of the ERK to SOS (via P90Rsk) negative feedback loop which rapidly disables the Ras pathway, whereas there is no feedback loop within the Rap1 pathway which leaves it free to be utilised by mutated EGFR. Therefore, this implies that in cancerous situations of EGFR mutation, drugs should target the C3G/Rap1/B-Raf pathway in order to effectively treat such cancers; furthermore, it also implies that drugs which target the SOS/Ras/Raf-1 pathway will be ineffective treatments.

**Figure 7:**
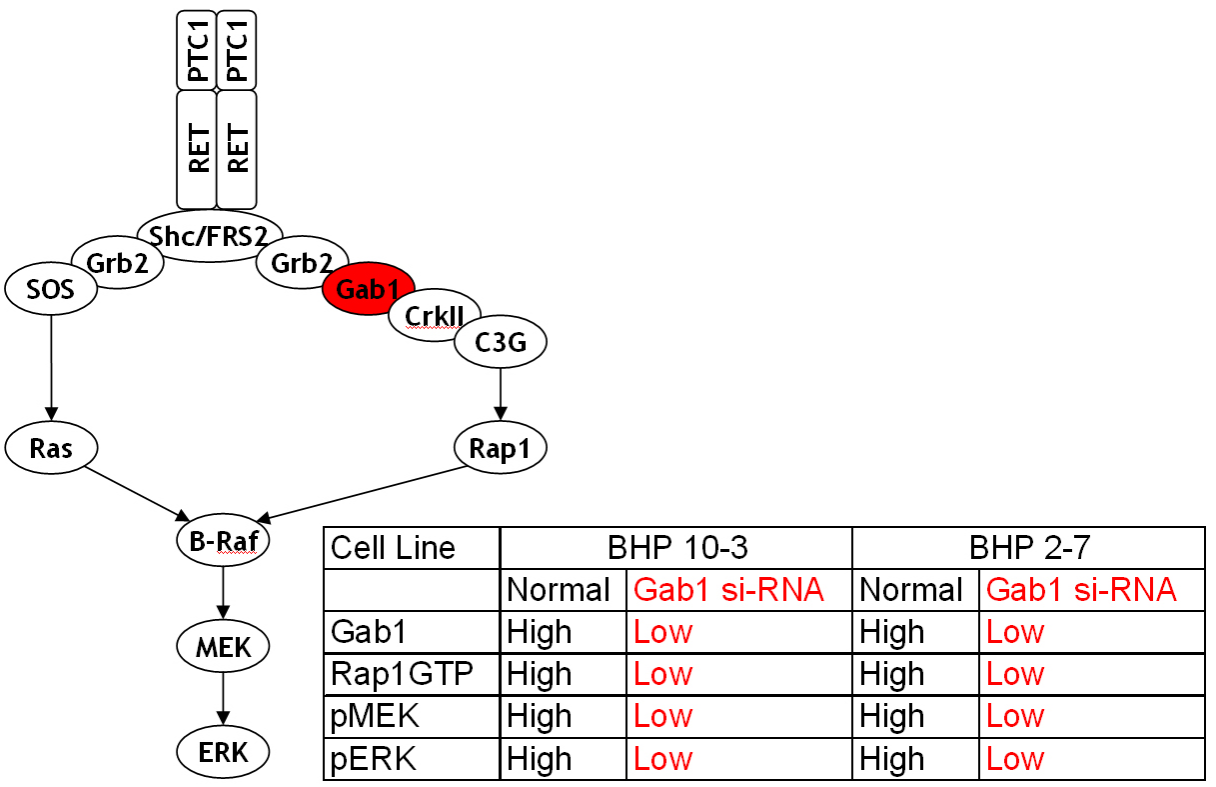
**Experimental results of Rap1 pathway knockout from Falco et al. (2007) **[37]: On the left, is a schematic of the mutated RET/PTC1 pathway as presented in [37]. On the right, is a table containing a summary of the results from the Gab1 knock down experiment using si-RNA in two different human PTC cell lines (BHP 10-3 and BHP 2-7) from [37] (please refer to Figure 5c in [37] for original blots); pMEK and pERK represent the activated phosphorylated forms of MEK and ERK, respectively, whilst Rap1GTP represents the activated GTP loaded form of Rap1.

To the best of our knowledge, our model prediction that the Rap1 pathway is the key pathway involved in signal transduction under EGFR mutation has not been reported previously. Indeed, it is the Ras pathway that has long been viewed as the critical pathway in EGFR signalling [9]. However, our model predicts that although the Ras pathway is indeed important for normal EGFR signalling, it is not important at all in signalling as the result of an EGFR mutation. To validate our model prediction, we searched through the scientific literature to try and find evidence of Rap1 activity in human cancers. We first found a paper by [36] who reported high levels of Rap1 activity in human metastatic melanomas and cutaneous metastatic melanoma tissues; increased ERK activity was also found in these tumours which interestingly harboured neither B-Raf nor Ras mutations. We subsequently found a recent study by [37] who investigated oncogenic signalling through the Rap1 pathway in human papillary thyroid carcinoma (PTC). PTCs feature chromosomal aberrations that result in the in-frame fusion of the intracellular kinase domain of the RET receptor with the NH2 terminus of heterologous proteins, generating the RET/PTC oncoproteins. If the RET receptor is fused with a protein partner that possesses a protein-protein interaction motif then it provides the RET/PTC kinases with a dimerising interface, thereby resulting in ligand-independent dimerisation, auto-phosphorylation and signalling; RET/PTC1 (the H4-RET fusion) and RET/PTC3 (the NCOA4-RET fusion) are the most prevalent variants. [37] firstly found high levels of Rap1 activation in human PTC cell lines which endogenously expressed RET/PTC1; these cells represent natural cancerous cells rather than artificially created cancerous cells generated through overexpression experiments. Secondly, [37] showed that a RET/PTC1-Gab1-Crk-C3G complex (i.e. the Rap1 pathway) was stably present in these cell lines. Thirdly, using si-RNA techniques, [37] were able to knock down Gab1 expression which resulted in the suppression of both Rap1 and ERK activation (Figure 7). It is important to note that knocking down Gab1 would have had no detrimental effect on the Ras pathway which signals via SOS; in actual fact knocking down Gab1 should have a positive effect on the Ras pathway as Gab1 and SOS can compete for binding to Grb2 (Figure 7). In essence, [37] showed that it is the Rap1 pathway that is the critical pathway involved in PTC cancerous signalling and that when the Rap1 pathway is knocked out (whilst leaving the Ras pathway intact), ERK activity is abolished. Therefore, this firmly supports our model prediction that it is the Rap1 pathway that is the key pathway involved in signal transduction under a cancerous receptor mutation. It is important to note that although the study by [37] was concerned with the RET (which plays a critical role in renal development) rather than the EGF receptor, the two receptors are very similar as they are both ligand induced, transmembrane receptor tyrosine kinases which can bind the same variety of adaptor proteins leading to the activation of both Ras and Rap1 and subsequently to ERK activation. Furthermore, both receptors are well known oncoproteins when mutations/deletions/fusions result in constitutively activated receptors that are capable of constitutively activating ERK. Therefore, we firmly believe that the findings by [37] regarding the RET receptor system are applicable to the EGFR system.

Our final model alteration was to investigate the effects of overexpressing the EGFR which is also a known cause of cancer. To accomplish this, the rate of receptor production was increased 100 fold to represent the increased transcription and translation of receptors. Rather than directly representing random receptor dimerisation, we simply stimulated the overexpressed receptors with EGF and analysed the differences between the normal and overexpressed systems; this is akin to the experimental strategy employed by [10] who ovexpressed EGFR in PC12 cells and subsequently stimulated them with EGF to investigate the effects of overexpression. The results from this experiment were extremely similar to those obtained in the EGFR mutation experiment, and therefore the simulation plots have been moved to Additional file 2 (Figure S4) as they are almost identical to the plots in Figure 6a and 6b. The overexpressed system resulted in the constitutive activation of ERK because the increased rate of receptor production counteracted the activated receptor degradation pathway, which resulted in a stable level of activated receptors on the cell membrane and the constitutive activation of the ERK pathway. Similar to the mutated EGFR receptors, the overexpressed receptors signalled predominantly via the C3G/Rap1/B-Raf as knocking out Ras or Raf-1 had little effect on the constitutive activation of ERK, whereas knocking out Rap1 of B-Raf had dramatic effects with active ERK levels falling to almost basal levels. Again, this was found to be due to the ERK to SOS (via P90Rsk) negative feedback loop shutting down the Ras pathway, and therefore implies that in cancerous situations of EGFR overexpression as well as EGFR mutation, drugs should target the Rap1 rather than Ras pathway.

Our models predict that the oncogenic EGFR signal passes almost exclusively via the Rap1 pathway and therefore predicts that drugs must target this pathway in order to effectively treat such cancers. Furthermore, our models predict that the key factor in oncogenic EGFR signalling is the ability to bypass or compensate for receptor degradation. When the receptor is overexpressed, activated receptors are still degraded but they are being constantly replenished due to the overexpression which means that there is a consistently high number of activated receptors on the membrane capable of constitutively activating the Rap1 pathway and therefore ERK. Whereas, mutated EGFR are not recognised as being active by the normal cellular machinery and are therefore not degraded which means that they too can constitutively activate the Rap1 pathway and ERK. Therefore, our model suggests that a drug capable of increasing the rate of EGFR degradation or capable of somehow tagging mutated EGFRs for degradation could also be a useful developments in cancer treatment.

### Comparison of Growth Factor Signalling

As described previously, our normal EGF model predicts that receptor degradation is the key process involved in signal termination and achieving only a transient activation of ERK after EGF stimulation; if the process of receptor degradation is deleted from the EGF model, a sustained ERK signal is observed (Figure 4c) and the sensitivity analysis identified it as one of the most sensitive reactions (see Additional file 2, Figure S6). This in itself is a novel prediction from our model, which to the best of our knowledge has not been reported previously. Indeed, most existing computational models of the EGF activated ERK pathway, including the original Brown model, do not include receptor degradation [11,29,38] or predict that it is not necessary for signal termination [28,33]. However, this also has wider implications with respect to ERK signalling from different growth factor receptors, and in particular could explain the differences between EGF and NGF signalling. In PC12 cells, EGF stimulates a transient activation of ERK whereas NGF stimulates a sustained activation of ERK, but it is still currently unclear how these different ERK signal dynamics can be robustly controlled by different upstream receptors utilising the same pathway. One of the known differences between the EGF and the NGF receptor systems is that whilst the EGFR is rapidly degraded, the NGF receptor TrkA is not degraded and remains active [9]. Our model predicts that it is this difference in receptor degradation that is the key difference between the two receptor systems. This can be investigated further by deleting the process of receptor degradation from our EGF model to generate a realistic model of the NGF receptor TrkA system.

**Figure 8:**
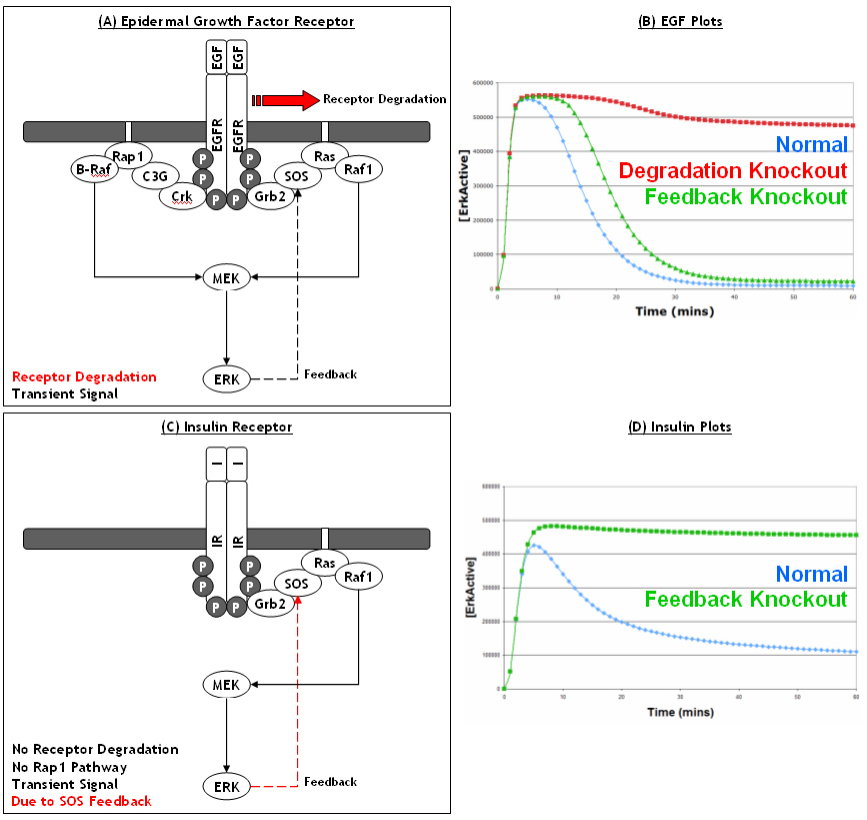
**Comparison of growth factor receptor signalling**: This figure shows a comparison of ERK signalling from the EGF and Insulin receptors. At the top, a schematic of the EGF induced ERK pathway is shown in (A), with receptor degradation highlighted in red as it is the key process in generating a transient response, this is demonstrated in (B). All the lines in (B) represent simulated active ERK levels over 60 minutes. The blue line represents active ERK levels from the normal EGF model, the green line represents active ERK levels when the SOS negative feedback loop is knocked out, whereas the red line represents active ERK levels when receptor degradation is knocked out. At the bottom, a schematic of the Insulin induced ERK pathway is shown in (C), with the SOS feedback loop highlighted in red as it is the key process in generating a transient response, this is demonstrated in (D). All the lines in (D) represent simulated active ERK levels over 60 minutes. The blue line represents active ERK levels from the normal Insulin model, whilst the green line represents active ERK levels when the SOS negative feedback loop is knocked out. It should be noted that although not shown, both the EGF and Insulin receptor models do include the PI3K-Akt pathway.

**Figure 9:**
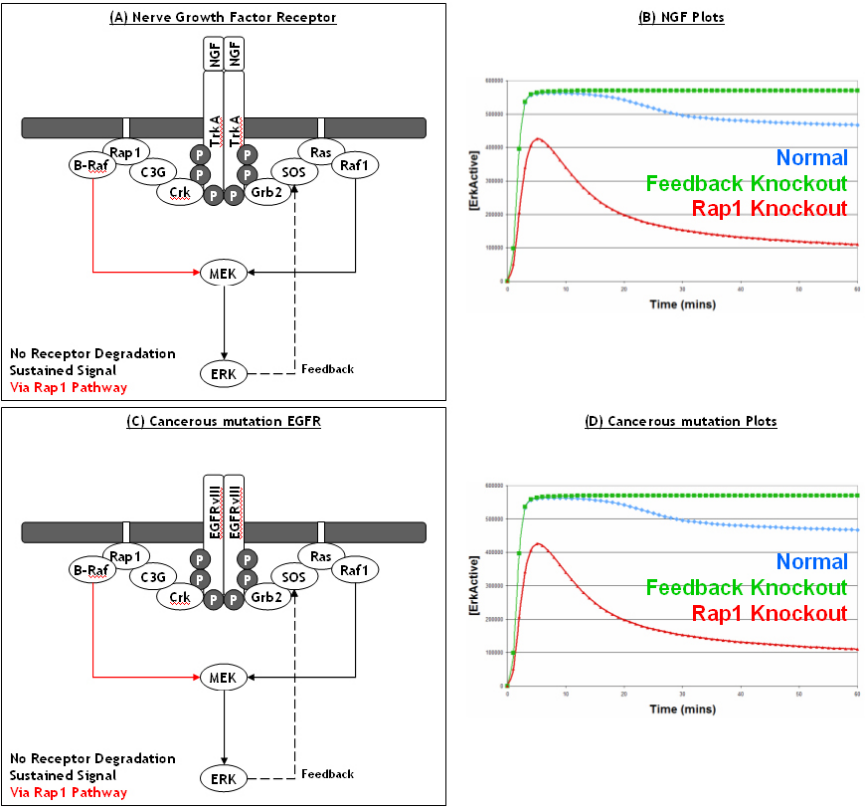
**Comparison of growth factor receptor signalling continued**: This figure shows a comparison of ERK signalling from the NGF and mutated EGF receptors. At the top, a schematic of the NGF induced ERK pathway is shown in (A), with the Rap1 pathway highlighted in red as it is the key process in generating a sustained response, this is demonstrated in (B). All the lines in (B) represent simulated active ERK levels over 60 minutes. The blue line represents active ERK levels from the normal NGF model, the green line represents active ERK levels after the SOS negative feedback loop is knocked out, whereas the red line represents active ERK levels after the Rap1 pathway is knocked out. Almost identical simulation results are found for the mutated EGFR model (C, D), where the Rap1 pathway is again found to be the key process in generating a sustained signal due to the lack of receptor degradation. It should be noted that although not shown, both the NGF and mutated EGF receptor models do include the PI3K-Akt pathway.

Another receptor that utilises the ERK pathway to relay its signal is the Insulin receptor. What is interesting about the Insulin receptor is that it shares properties with both the EGF and NGF receptor systems. Like the EGF receptor, the Insulin receptor generates a transient activation of ERK; EGF stimulates cells to proliferate whereas insulin primarily has metabolic effects. However, unlike the EGF receptor, the Insulin receptor is not degraded as it is efficiently recycled back to the plasma membrane. This would appear to contradict our previous prediction that receptor degradation is the key to achieving a transient response, and leads to the question as to how the Insulin receptor can generate only a transient ERK response without the process of receptor degradation. However, one explanation for this could be that the Insulin receptor system is not able to utilise the Rap1 pathway [4,33]. To investigate further we are able to generate a realistic model of the Insulin receptor system by deleting both receptor degradation and the Rap1 pathway from the EGF model. Therefore, we now have 3 models of 3 different receptor systems (EGF, NGF, Insulin) to directly compare and contrast, as well as the models of the cancerous EGFR system (Figures 8, 9).

As can be seen in Figures 8, the process of receptor degradation is essential to achieve a transient response in the EGFR system whilst the SOS feedback loop is redundant, as removing it has little effect on the transient ERK signal dynamics (Figure 8A, B). In contrast, the SOS feedback loop is essential to achieve a transient response in the Insulin system (Figure 8C, D). This is because Insulin receptors are not degraded, therefore the only way to achieve a transient signal is via the SOS feedback loop to shutdown the Ras pathway. Another key to the transient signal achieved via Insulin stimulation, is the fact that the Insulin receptor is unable to utilise the Rap1 pathway, which does not contain a feedback loop. If the Insulin receptor were able to utilise this pathway a sustained signal would surely be observed. Indeed, as can be seen for the NGF receptor TrkA a sustained ERK signal is indeed observed as the TrkA receptor is not degraded and is able to utilise the Rap1 pathway (Figure 9A, B). One interesting point, is that the NGF receptor system is in fact very similar to the mutated EGFR system as both systems contain receptors that are not degraded, both can utilise the Ras and Rap1 pathways, and both result in a sustained ERK signal (Figure 9C, D). As discussed previously, the key to cancerous signalling from the EGF receptor is the ability to bypass or compensate for the process of receptor degradation. In the case of overexpressed EGFR, the process of receptor degradation is compensated for by the increased rate of production of new EGF receptors. Combining all of the above leads to the prediction that, if the only real difference between EGF and NGF receptors is indeed receptor degradation, a PC12 cell that is stimulated with EGF and has overexpressed EGFR should result in a sustained ERK signal and neuronal differentiation, not cellular proliferation. Interestingly, this prediction from our model has already been validated in a seminal experimental by [10] who overexpressed EGFR in PC12 cells and observed sustained ERK as well as neuronal differentiation. Therefore, this further backs up our models and predictions about receptor degradation being a critical process and a key difference between the receptor systems.

In summary, the critical difference between the EGF and the NGF systems is receptor degradation, with the EGF receptor being rapidly degraded after stimulation and therefore only generating a transient ERK signal, whilst the NGF receptor TrkA is not degraded and generates a sustained ERK signal. Whereas, the critical difference between the Insulin and the NGF systems (both of which are not degraded) is the Rap1 pathway, with the NGF receptor TrkA being able to utilise it to generate a sustained ERK signal, whilst the Insulin receptor is unable to utilise it and can only use the Ras pathway which is rapidly shutdown via the SOS feedback loop and therefore only generate a transient ERK signal. As the insulin receptor is not degraded, this makes the SOS feedback loop essential for signal termination and generating only a transient ERK signal [4]. Whereas, as the EGF receptor is degraded, the SOS feedback loop is redundant under EGF signalling and not required for signal termination. Overall, our results suggest that the ERK pathway has evolved to be utilised by numerous upstream receptors and that the differences between ERK signalling from different growth factor receptors seem to be large scale, with entire processes (such as receptor degradation) or entire pathways (such as the Rap1 pathway) being either present or absent, rather than subtle differences in the kinetics of protein binding or activation.

In our previous work, we already showed through modelling and experimental validation that the SOS feedback loop was not required for efficient signal termination and the transient activation of ERK induced by EGF [33]. However, as we did not consider the Rap1 pathway in this model, we incorrectly hypothesised that receptor degradation could also be redundant as the SOS feedback loop could compensate for its absence. We drew parallels to the Insulin receptor system which lacked receptor degradation, and where the SOS feedback loop had previously been shown experimentally to be essential for signal termination and generating a transient ERK response [4,33]. Although the work presented here does not affect our previous conclusions, it does take our previous work another step forward by considering the Rap1 pathway to show that although the SOS feedback loop is indeed redundant, the process of receptor degradation is actually essential in EGF signalling. This highlights the fact that models, like our biological understanding of the pathway itself, can evolve over time and be used to suggest interesting new hypotheses and explanations for the observed data that challenge our current understanding.

## Conclusion

In this study, we used computational modelling techniques to investigate what effects various cancerous alterations had on signalling through the EGF activated ERK pathway. We initially introduced a number of cancerous mutations into the original EGF model developed by [29] but found that the model incorrectly predicted the effects of an EGFR mutation due to the fact that the model was incomplete (Figure 2b). We therefore constructed a new model of the EGF activated ERK pathway by taking the original Brown EGF model and expanding it to include receptor production and degradation as well as the C3G/Rap1/B-Raf pathway, which we believed were important processes in both normal EGF and cancerous signalling. This model expansion was informed by the scientific literature, and in particular the study by [9] which is one of the most comprehensive and up-to-date studies of the EGF and NGF activated ERK pathway. We used our new model to investigate the effects of cancerous mutations and what the best drug targets would be, as well as using the model to conduct a comparison of different growth factor receptors, and as a result we have generated a number of interesting and novel predictions.

Our model predicts that the oncogenic EGFR signal passes almost exclusively via the Rap1 pathway and therefore predicts that drugs must target this pathway in order to effectively treat such cancers. Furthermore, our models predict that the key factor in oncogenic EGFR signalling is the ability to bypass or compensate for receptor degradation. When the receptor is overexpressed, activated receptors are still degraded but they are being constantly replenished due to the overexpression which means that there is a consistently high number of activated receptors on the membrane capable of constitutively activating the Rap1 pathway and therefore ERK. Whereas, mutated EGFR are not recognised as being active by the normal cellular machinery and are therefore not degraded which means that they too can constitutively activate the Rap1 pathway and ERK. Therefore, our model suggests that a drug capable of increasing the rate of EGFR degradation or capable of somehow tagging mutated EGFRs for degradation could be a useful developments in cancer treatment.

Our model predicts that normal EGFR signalling results in a transient ERK signal due to receptor degradation, NGF signalling results in a sustained ERK signal (via the Rap1 pathway) as there is no degradation of the TrkA receptor, and cancerous EGFR signalling results in a constitutive/sustained ERK signal (via the Rap1 pathway) because receptor degradation is either abolished or counteracted (Figure 8). Overall, this highlights the importance of the Rap1 pathway in both normal and oncogenic EGFR signalling as well as in NGF signalling. The key feature of the Rap1 pathway is that it lacks a negative feedback loop, and will therefore keep signalling if the receptor remains active; however, it should be noted that although there is no negative feedback loop currently known, one can never rule out the possibility of one being identified in the future. Furthremore, our models predict that the key difference between the EGF and NGF receptor systems is receptor degradation. The behaviour of these two receptor systems in PC12 cells has long fascinated many researchers and our simple prediction appears to be both novel and effectively explain how the two receptor systems are able to utilise the same pathway to achieve different cellular responses. Interestingly, the ERK to SOS (via P90Rsk) negative feedback loop appears to be irrelevant in both EGF and NGF signalling, therefore one may wonder why the SOS feedback loop is even there. However, the SOS feedback loop is essential for a transient ERK response to be achieved in systems such as the insulin receptor, which are not degraded and can not utilise the Rap1 pathway (Figure 8). Overall, our results suggest that the ERK pathway has evolved to be utilised by numerous upstream receptors and that the differences between ERK signalling from different growth factor receptors seem to be large scale, with entire processes (such as receptor degradation) or entire pathways (such as the Rap1 pathway) being either present or absent, rather than subtle differences in the kinetics of protein binding or activation. Therefore, as the differences between receptor systems are essentially structural, this could suggest that more qualitative modelling techniques such as Petri-Nets [39,40] or logical process algebras [41,42], which are more traditional tools for analysing model structure, could play important roles in the analysis and comparison of signal transduction pathways. Furthermore, as the different growth factor receptor systems appear to be so similar, this suggests that a model of one growth factor receptor systems could be readily applied to another receptor system with relatively simple modifications. Indeed, in this study we created a model of the NGF receptor based on the EGF receptor model as well as drawing comparisons to the RET receptor, and previously we created a model of the insulin receptor based on a model of the EGF receptor [33]. However, the ERK pathway is not the only pathway initiated by growth factor receptors and as different receptors eventually lead to different biological responses, models will need to evolve in the future to include these alternative adaptor proteins and pathways, and ultimately their influence on gene expression.

A recent study by [12] suggested that a critical difference between the EGF and NGF systems was that a negative feedback loop existed between Raf-1 and ERK under EGF, but under NGF this was transformed into a positive feedback loop resulting in the sustained activation of ERK via Raf-1. However, studies by [9,43], which we have used to inform our model, showed that the sustained ERK signal from NGF stimulation is a result of Rap1/B-Raf activity, and that Ras/Raf-1 is only used transiently under NGF. The EGF vs NGF phenomenon in PC12 cells has long fascinated many researchers and has been well studied, but it is still unclear exactly how different ERK signal dynamics can be robustly controlled by different upstream receptors utilising the same pathway, especially given such differing data. It is important to note here, that we are not implying that our model and the data we have used should be trusted more than any others. Rather we are implying that our model offers an interesting and alternative explanation for the observed data, we have been able to expand our model out from the traditional EGF vs NGF system to incorporate cancerous mutations as well as other growth factor receptors, and importantly we have made a number of interesting predictions which we have been able to validate through existing experimental results in the scientific literature. Only time and further laboratory data can tell which models and hypotheses are truly correct, if any, but it is important to remember that all models are simplifications of the true real-life situation, and therefore any predictions from them should be treated with some caution. In the words of George E. P. Box, "Essentially, all models are wrong, but some are useful" [44].

In conclusion, this study has shown how computational models can be useful tools for investigating and comparing the biological behaviour of signal transduction pathways as they can suggest new hypotheses to explain the observed biological data and help understand the dynamics of how the pathway functions. Furthermore, computational models can be readily used to investigate different disease states and suggest how drug treatment could be improved to better combat the effects of the disease. Ultimately, the behaviour of computational models needs to be validated with experimental data from the laboratory so that any predictions made from them can be trusted. Therefore, we have validated the behaviour of our model with our own as well as published experimental data and have found supporting evidence for our predictions in the scientific literature. We therefore believe that our model is a good representation of the EGF activated ERK pathway which can be expanded and applied in the future to further investigate the dynamics and functioning of growth factor receptor signalling.

## Authors' contributions

RJO carried out the modelling and analysis work, with initial investigations into cancerous mutation done by MEA. AG and OS carried out the experimental laboratory work. All of the work was supervised by DRG and WK. This work was carried out whilst RJO, MEA, AG, OES and DRG were working at the Bioinformatics Research Centre, Department of Computing Science, University of Glasgow.

## Supplementary Material

Additional file 1 **EGF Model**. This is an SBML file of our newly developed EGF model Click here for file

Additional file 2 **Additional Information and Figures**. This word file contains additional information such as laboratory protocols, details on how all the model knockouts were performed, model sensitivity analysis results, and additional figures. Click here for file

Additional file 3 **Model Parameters**. This excel file contains the values and sources of all the model parameters - specie initial concentrations and rate constants. Click here for file
