# Supplementary material for: Computational modelling of cancerous mutations in the EGFR/ERK signalling pathway
Source: BMC Syst Biol. 2009 Oct 5;3:100. doi: 10.1186/1752-0509-3-100 (PMC2764635; doi:10.1186/1752-0509-3-100)
Supplement: Additional file 2 — Additional Information and Figures. This word file contains additional information such as laboratory protocols, details on how all the model knockouts were performed, model sensitivity analysis results, and additional figures. [file 1752-0509-3-100-S2.DOC]

**Additional Information**

**S1: Cell culture**

PC12 cells were maintained in Dulbecco’s modified Eagle’s minimal (DMEM) medium (Sigma) supplemented with 10 % (v/v) horse serum, 5 % (v/v) newborn calf serum (Sigma), 1% (v/v) glutamax, 100 U/mL penicillin and 100 µg/mL streptomycin (Gibco BRL, Paisley, UK) at 5% CO_2_. For treatment with EGF, PC12 cells were plated on poly-lysine coated 6-well plates at 50 – 60 % confluency and deprived of serum for 8 hours in DMEM medium supplemented with 1% (v/v) glutamax, 100 U/mL penicillin and 100 µg/mL streptomycin. Then, EGF (Promega) was added to a final concentration of 50 ng/ml and the cells were incubated at 37ºC, 5% CO_2_ for the desired time interval. The treatment was stopped by washing the cells with ice-cold PBS and immediate flash-freezing in dry ice. To inhibit MEK1/2, U0126 (Cell Signalling Technologies) was added to a final concentration of 25µM 1 hour prior to EGF-treatment.

**S2: Western blotting**

Cells were lysed at 4ºC in 3T3-cell lysis buffer (25mM HEPES pH 7.4, 50mM NaCl, 5mM EDTA, 1% Triton X-100) supplemented with protease inhibitors (Protease Inhibitor cocktail, Roche) and phosphatase inhibitors (2mM NaF, 0.2mM NaP_2_O_5_, 0.5mM sodium orthovanadate, 10mM β-glycerophosphate). Lysates were cleared by centrifugation at 16,000xg at 4ºC for 15 minutes. Protein concentrations were quantified by a modified Lowry assay (Bio-Rad Dc protein assay; Bio-Rad Laboratories). The samples were separated by sodium dodecylsulfate – polyacrylamide gel electrophoresis (SDS-PAGE) using NOVEX 4-12% BisTris gradient gels (Invitrogen) and transferred to nitrocellulose membranes (Schleicher and Schuell). After blocking with TBS-Tween buffer (150mM NaCl, 50mM TrisHCl pH 7.4, 0.1% Tween-20) containing 5% nonfat dry milk for 1 h at room temperature, the membranes were probed with primary phosho-ERK1/2 (1:1000), and ERK1/2 (1:1000) (Cell Signalling Technologies) diluted in TBS-Tween plus 5% nonfat dry milk, as indicated. The phospho-specific antibodies specifically detect the activating phosphorylations and therefore can be used to assess activation of ERK. The blots were then incubated with the appropriate secondary antibodies conjugated with LI-COR IRDye© 680 nm and the band intensities were quantified by immunofluorescence using an Odyssey scanner (LICOR). Total amounts of ERK remained constant under these experimental conditions.

**S3: Simulation Results from Ras mutation model with Raf-1 and B-Raf**


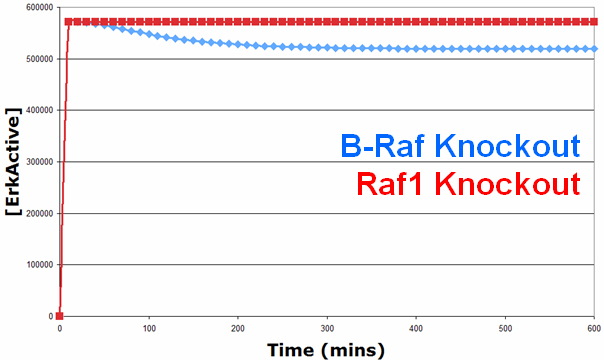


The red and blue lines represent the simulated levels of active ERK over 600 minutes with a Ras mutation. and a Raf1 or B-Raf knockout, respectively. As can be seen, under the Ras mutation, knocking out Raf1 or B-Raf individually has little effect on the ERK signal, with active ERK levels remaining very high. This therefore suggests that drugs attempting to treat cancers caused by Ras mutation, will need to target both the Raf isoforms in order to be effective treatments.

**S4: Simulation results from EGFR overexpression model**

**
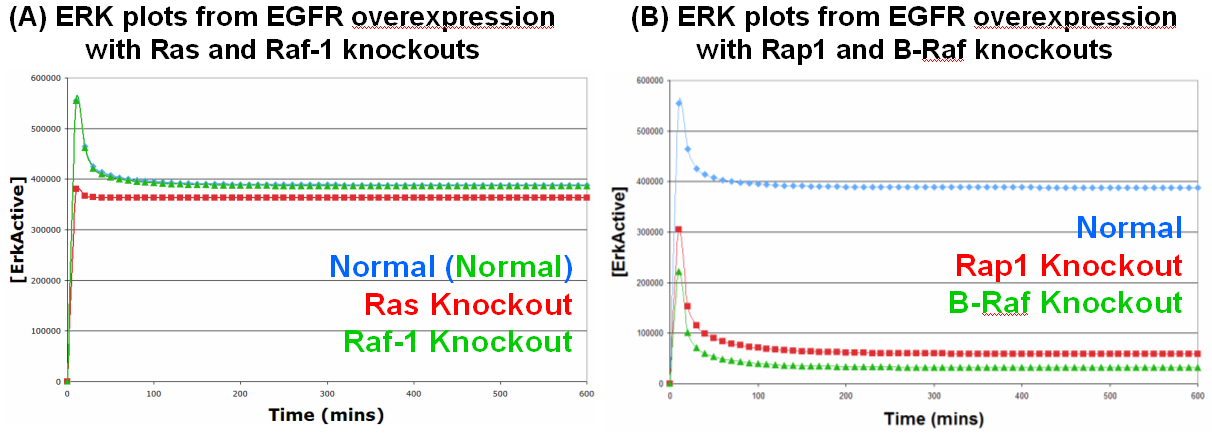
**

(A) Ras and Raf-1 Knockouts: All the lines on this graph represent simulated active ERK levels over 600 minutes. The blue line represents active ERK levels with EGFR overexpression, whereas, the red and green lines represent active ERK levels with EGFR overexpression but also with a Ras or Raf-1 knockout, respectively; the green line is practically identical to the blue line and hence obscures the blue line from view. As can be seen, introducing EGFR overpression into the model results in the sustained activation of ERK, as expected. However, knocking out Raf-1 has no effect on the ERK signal whereas knocking out Ras has only a slight effect, suggesting that the cancerous signal passes via an alternative route. (B) Rap1 and B-Raf Knockouts: All the lines on this graph represent simulated active ERK levels over 600 minutes. The blue line represents active ERK levels with EGFR overexpression (the same as the blue line in graph A), whereas, the red and green lines represent active ERK levels with EGFR overexpression but also with a Rap1 or B-Raf knockout, respectively. As can been seen, knocking out Rap1 or B-Raf have dramatic effects on the ERK signal with activated ERK levels falling to near basal levels. Overall, these results suggest that overexpressed EGFR signal almost exclusively via the C3G/Rap1/B-Raf pathway and that the SOS/Ras/Raf-1 pathway is hardly used due to the SOS negative feedback loop shutting the pathway down.

**S5: Model Mutation Descriptions**

- Figure 2B: EGFR Mutation: In the original Brown model, the species EGF, freeEGFReceptor, and boundEGFReceptor were deleted along with the EGF-EGFR binding and unbinding reactions. A new species called mutatedEGFReceptor was then introduced with an initial concentration of 80,000 (which was the initial concentration of freeEGFReceptor). The Sos Activation reaction was then changed so that the new catalyst is mutatedEGFReceptor rather than the deleted boundEGFReceptor species. Thus creating a constitutively active EGFR species.
- Figure 2B: Ras Mutation: In the original Brown model, the concentration of EGF was set to 0 to disable receptor activation, the species RasInactive and RasActive were then deleted along with the Ras Activation (by Sos) and Ras Deactivation (by RasGAP) reactions, and a new species RasMutated was introduced with an initial concentration of 120,000 (which was the initial concentration of RasInactive). The Raf1 and PI3K activation reactions were then changed so that the new catalyst is RasMutated rather than the deleted RasActive. Thus creating a constitutively active Ras species.
- Figure 2C: Akt Knockout: In the original Brown model, the initial concentration of AktInactive is set to 0, thus knocking out all Akt species in the model, and disabling the Akt-Raf1 negative feed-forward loop.
- Figure 2C: Sos Knockout: In the original Brown model, the initial concentration of P90RskInactive is set to 0, thus knocking out all P90Rsk species in the model, and disabling the Sos negative feedback loop.
- Figure 4C: Degradation Knockout: The EGFReceptor_Degradation reaction is simply deleted from the model.
- Figure 4C: Feedback Knockout: As before, the initial concentration of P90RskInactive is set to 0, thus knocking out all P90Rsk species in the model, and disabling the Sos negative feedback loop.
- Figure 5A: Ras Mutation: The concentration of EGF was set to 0 to disable receptor activation, the species RasInactive and RasActive were then deleted along with the Ras Activation (by Sos) and Ras Deactivation (by RasGAP) reactions, and a new species RasMutated was introduced with an initial concentration of 120,000 (which was the initial concentration of RasInactive). The Raf1, bRaf and PI3K activation reactions were then changed so that the new catalyst is RasMutated rather than the deleted RasActive. Thus creating a constitutively active Ras species.
- Figure 5B: B-Raf Mutation: The concentration of EGF was set to 0 to disable receptor activation, the species bRafInactive and bRafActive were then deleted along with the bRaf1 Activation (by Rap1), bRaf1 Activation (by Ras), and bRaf1 Deactivation (by RafPPtase) reactions, and a new species bRafMutated was introduced with an initial concentration of 120,000 (which was the initial concentration of RasInactive). The Mek Activation reaction was then changed so that the new catalyst is bRafMutated rather than the deleted bRafActive. Thus creating a constitutively active bRaf species.
- Figure 6A: EGFR Mutation with Ras Knockout: The species EGF, freeEGFReceptor, and boundEGFReceptor were deleted along with the EGF-EGFR binding and unbinding reactions, and the EGFR production and degradation reactions. A new species called mutatedEGFReceptor was then introduced with an initial concentration of 80,000 (which was the initial concentration of freeEGFReceptor). The Sos and C3G Activation reaction were then changed so that the new catalyst is mutatedEGFReceptor rather than the deleted boundEGFReceptor species. Thus creating a constitutively active EGFR species. The Ras Knockout was achieved by setting the initial concentration of RasInactive to 0 thus knocking out all Ras species in the model.
- Figure 6A: EGFR Mutation with Raf-1 Knockout: EGFR mutation as above, Raf1 Knockout was achieved by setting the initial concentration of Raf1Inactive to 0 thus knocking out all Raf1 species in the model.
- Figure 6B: EGFR Mutation with Rap1 Knockout: EGFR mutation as above, Rap1 Knockout was achieved by setting the initial concentration of Rap1Inactive to 0 thus knocking out all Rap1 species in the model.
- Figure 6B: EGFR Mutation with B-Raf Knockout: EGFR mutation as above, B-Raf Knockout was achieved by setting the initial concentration of B-RafInactive to 0 thus knocking out all B-Raf species in the model.
- Figure S3: Ras Mutation introduced as in Figure 5A above. Raf1 and B-Raf1 knockouts achieved by setting the initial concentration of Raf1Inactive and B-RafInactive, respectively, to 0 thus knocking out all forms of the corresponding Raf species from the model.
- Figure S4: EGFR Overexpression with Ras, Raf1, Rap1, and B-Raf Knockouts: the rate of receptor production was increased 100 fold to represent the increased transcription and translation of receptors. Mutations of Ras, Rap1, Raf1 and B-Raf achieved as in Figure 6A and 6B above.

**S6: Sensitivity Analysis**

Sensitivity analysis is a general technique for establishing the contribution of individual parameter values to the overall performance of a complex system. To accomplish this, we first simulated the model under normal parameter conditions for 60 minutes, monitoring the concentration of activated ERK at each minute giving 60 data points. We then calculated the area underneath the activated ERK curve by simply summing up all the data points. We then took each reaction rate parameter in turn and decreased it by 10%, re-simulated the model, and re-calculated the area underneath the activated ERK curve. The sensitivity (S) of the system to the change in the parameter (P) with respect to our selected system output, activated ERK (E), could then be calculated as S = (ΔE/E) / (ΔP/P) where ΔE is the change in E caused by the change in P (ΔP).

As can be seen, reactions contained within the Rap1 pathway were found to be more sensitive than the corresponding reactions in the Ras pathway. This is to be expected given that the Ras pathway is contained within a strong negative feedback loop, thus reducing the sensitivities of the reactions contained within the loop. However, although less sensitive, the Ras pathway is still a key feature of the EGFR system. This is illustrated in the knockout plots in Figure 4d, as knocking out Ras has a greater effect on the peak of the ERK signal than knocking out Rap1. This again highlights the fact that the normal EGFR system utilises both of the pathways to relay its signal. The sensitivity analysis also highlighted EGF receptor degradation as one of the most sensitive reactions in the model. This further highlights the importance of the process of receptor degradation in addition to the knockout experiments in Figure 4c.

| **No** | **Reaction Name** | **Parameters** | **Sensitivity** |
| --- | --- | --- | --- |
| 8 | bRaf_Deactivation | kcat | -0.884547058 |
| 15 | EGFReceptor_Degradation | k1 | -0.604191942 |
| 44 | Rap1_Deactivation | kcat | -0.600987857 |
| 26 | Mek_Deactivation | kcat | -0.521853955 |
| 20 | Erk_Deactivation | kcat | -0.492486829 |
| 48 | Ras_Deactivation | kcat | -0.311397705 |
| 12 | C3G_Deactivation | k1 | -0.292989797 |
| 11 | C3G_Activation | km | -0.282753052 |
| 25 | Mek_Activation_bRaf | km | -0.250044673 |
| 19 | Erk_Activation | km | -0.24176474 |
| 5 | bRaf_Activation | km | -0.206278522 |
| 51 | Sos_Activation | km | -0.156510968 |
| 43 | Rap1_Activation | km | -0.139840031 |
| 7 | bRaf_Activation_Ras | km | -0.101615852 |
| 53 | Sos_Feedback_Deactivation | kcat | -0.094063538 |
| 28 | P90Rsk_Activation | kcat | -0.091493258 |
| 47 | Ras_Activation | km | -0.073751881 |
| 52 | Sos_Deactivation | k1 | -0.062649112 |
| 38 | Raf1_Deactivation | kcat | -0.02409646 |
| 23 | Mek_Activation | km | -0.017202378 |
| 37 | Raf1_Activation | km | -0.008606118 |
| 40 | Raf1_Deactivation_Akt | kcat | -0.001574188 |
| 1 | Akt_Activation | kcat | -0.001476167 |
| 31 | PI3K_Activation_EGFR | kcat | -0.000103327 |
| 13 | EGF_Binding_Unbinding | k1 | -5.29376E-06 |
| 16 | EGFReceptor_Degradtion_Free | k1 | -2.94373E-06 |
| 33 | PI3K_Activation_Ras | kcat | -2.7582E-06 |
| 34 | PI3K_Activation_Ras | km | -2.7582E-06 |
| 14 | EGF_Binding_Unbinding | k2 | 6.27088E-06 |
| 35 | PI3K_Deactivation | k1 | 1.44218E-05 |
| 3 | Akt_Deactivation | k1 | 6.8992E-05 |
| 32 | PI3K_Activation_EGFR | km | 7.53E-05 |
| 2 | Akt_Activation | km | 0.001326161 |
| 30 | P90Rsk_Deactivation | k1 | 0.001673595 |
| 41 | Raf1_Deactivation_Akt | km | 0.001695215 |
| 39 | Raf1_Deactivation | km | 0.015095042 |
| 22 | Mek_Activation | kcat | 0.017442131 |
| 36 | Raf1_Activation | kcat | 0.023082025 |
| 17 | EGFReceptor_Production | k1 | 0.050111092 |
| 29 | P90Rsk_Activation | km | 0.077651501 |
| 54 | Sos_Feedback_Deactivation | km | 0.089465105 |
| 50 | Sos_Activation | kcat | 0.157400295 |
| 9 | bRaf_Deactivation | km | 0.2265377 |
| 24 | Mek_Activation_bRaf | kcat | 0.270077667 |
| 6 | bRaf_Activation_Ras | kcat | 0.279306729 |
| 10 | C3G_Activation | kcat | 0.279424812 |
| 49 | Ras_Deactivation | km | 0.299246884 |
| 46 | Ras_Activation | kcat | 0.301324399 |
| 18 | Erk_Activation | kcat | 0.307576897 |
| 27 | Mek_Deactivation | km | 0.404704483 |
| 21 | Erk_Deactivation | km | 0.430817668 |
| 45 | Rap1_Deactivation | km | 0.546709662 |
| 42 | Rap1_Activation | kcat | 0.550755133 |
| 4 | bRaf_Activation | kcat | 0.560722922 |

**S7: Additional Files**

- An electronic SBML file of our full EGFR model has been submitted as Supplementary Information along with the manuscript
- An electronic Excel file giving details of all model parameters and their sources has been submitted as Supplementary Information along with the manuscript
